# Confinement induces internal flows in adherent cell aggregates

**DOI:** 10.1098/rsif.2024.0105

**Published:** 2024-05-22

**Authors:** M. S. Yousafzai, S. Amiri, Z. G. Sun, ‪A. A. Pahlavan, M. Murrell

**Affiliations:** ^1^ Department of Biomedical Engineering, Yale University, , CT 06511, USA; ^2^ Systems Biology Institute, Yale University, CT 06516, USA; ^3^ Department of Mechanical Engineering and Materials Science, Yale University, , CT 06511, USA; ^4^ Department of Physics, Yale University, , CT 06511, USA

**Keywords:** convection, confinement, cell aggregates, traction force, surface tension, migration

## Abstract

During mesenchymal migration, F-actin protrusion at the leading edge and actomyosin contraction determine the retrograde flow of F-actin within the lamella. The coupling of this flow to integrin-based adhesions determines the force transmitted to the extracellular matrix and the net motion of the cell. In tissues, motion may also arise from convection, driven by gradients in tissue-scale surface tensions and pressures. However, how migration coordinates with convection to determine the net motion of cellular ensembles is unclear. To explore this, we study the spreading of cell aggregates on adhesive micropatterns on compliant substrates. During spreading, a cell monolayer expands from the aggregate towards the adhesive boundary. However, cells are unable to stabilize the protrusion beyond the adhesive boundary, resulting in retraction of the protrusion and detachment of cells from the matrix. Subsequently, the cells move upwards and rearwards, yielding a bulk convective flow towards the centre of the aggregate. The process is cyclic, yielding a steady-state balance between outward (protrusive) migration along the surface, and ‘retrograde’ (contractile) flows above the surface. Modelling the cell aggregates as confined active droplets, we demonstrate that the interplay between surface tension-driven flows within the aggregate, radially outward monolayer flow and conservation of mass leads to an internal circulation.

## 1. Introduction

Collective cell motion drives essential physiological processes, from the separation of tissues in early development [1–3] to the invasion of cells during the pathogenesis of cancer [4–6]. Collective motion is driven by a balance of mechanical forces exerted between cells and the extracellular matrix (ECM) and mechanical forces between cells [7,8]. These results have been predominantly limited to describing collective motion in quasi-two-dimensional tissues, such as cell monolayers [7–13]. While three-dimensional flows have been observed within developmental systems and model systems including organoids and cell aggregates [14–17], a mechanistic understanding of how traction forces coordinate with large-scale surface stresses and pressures in three-dimensional tissues remains unclear.

At the cellular level, forward mesenchymal migration is initiated by the assembly and protrusion of F-actin against the cell membrane [18]. F-actin protrusion, coupled with simultaneous engagement and contractility of F-actin by myosin molecular motors, determines the rate of F-actin ‘retrograde’ flow, across the lamella [19–21]. This F-actin flow, through frictional interactions with integrins within focal adhesions, generates a drag and external traction force against the ECM [22,23]. This frictional interaction reflects a ‘clutch’ between actin flow and traction force, yielding an inverse force–velocity relationship [24–28], and determines the rate of cell advance [20]. Thus, at the cellular level, migration can be described by a balance of protrusive and contractile interactions and frictional coupling between the cell and ECM. However, tissues may have strong surface stresses and high pressures [17,29–32] and thus may drive rapid and long-range motions through surface tension gradients or pressure gradients [14,15,32,33]. Thus, cell advance depends upon the generation and transmission of forces across diverse length scales.

The collective migration of cells within clusters or ‘aggregates’ has drawn comparisons to the physics of liquid droplets wetting hydrophilic surfaces [34–40]. By this analogy, the interactions between cells and the ECM are abstracted as interfacial tensions between the droplet and the surface. For strong cell–ECM tensions, cells protrude from the aggregate and migrate collectively outwards, expanding a monolayer. The monolayer expands until ultimately the aggregate is depleted of cells, and only a monolayer remains (i.e. fully wetting). By contrast, for weak cell–ECM tensions, dispersed cells can form an aggregate, i.e. de-wetting from the substrate [35,38,41,42]. The origin of de-wetting lies in the stresses transmitted via E-cadherin cell–cell contacts. Thus, to date, no steady state, controlled experimental condition exists, in which both the aggregate as well as its expanding monolayer coexist, reflecting a partially wetted state. As a result, the conditions under which protrusion (migration) and retraction (convection) are coordinated are unclear.

Here, we adhere and spread cell aggregates onto polyacrylamide (PAA) gels with and without adhesive micropatterns. Without micropatterns, we observe that aggregates are fully wet on the surface [15]. However, for micropatterns that are sufficiently small to prevent full wetting of the aggregate, we observe the coexistence of both monolayer and aggregate in a steady, pseudo-partially wetted state. In this case, the contact line is ‘pinned’. We then quantify the motion of cells within the monolayer (at the gel surface) which occurs owing to cell migration, and the motion of cells within the aggregate (above the gel surface), to which we draw analogies to a convective recirculating flow field. We contrast the extent that each of these motions is exhibited in both partially wetting and fully wetting states. To explain the differences in dynamics between these two states, we argue that the effect of confinement creates a differential balance of mechanical stresses exerted by monolayer expansion (traction) and surface stresses exerted by the aggregate. Our observations highlight the important role of confinement, not only in setting the extent of migration but also in creating bulk tissue-scale convective flows that determine the net dynamics of cellular assemblies.

## 2. Results

### 2.1. Adhesive confinement inhibits aggregate spreading and maintains a partially wetted state

**Figure 1.**
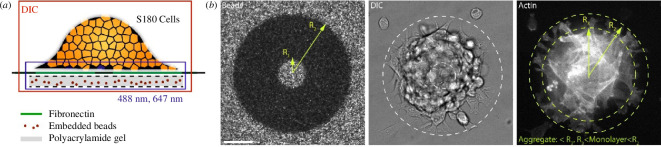
Confining adhesion maintains the aggregate and monolayer in a partially wet state. (*a*) Schematic of experiment. An S180 cell aggregate adheres to a fibronectin-coated (micropatterned) PAA gel, in which 40 nm beads have been embedded. They are imaged by transmitted light (DIC), as well as fluorescence (488 and 568 nm). (*b*, left) Fluorescence of beads embedded in an Y = 8.6 kPa PAA gel. Dark regions indicate activation areas for fibronectin attachment. Activation photo-bleaches the beads. Radii of micropatterns are indicated. (*b*, middle) DIC image of an S180 cell aggregate adherent to the fibronectin pattern. White-dashed circle indicates the region of the micropattern. (*b*, right) F-tractin image of the cell aggregate, visualizing cells at the confocal plane. Yellow-dashed circles indicate regions in which the aggregate is confined (between 0 and *R*_3_), and where single cells protrude but are excluded from the aggregate (beyond *R*_3_). Scale bar is 50 μm.

Cell aggregates adhere to ‘confined’ micropatterned adhesive (fibronectin) islands or ‘unconfined’ uniformly adhesive PAA gels. The confined pattern has an outer radius (*R*_2_) of 100 μm (later referred to as the ‘contact line’) and an inner radius (*R*_1_) of 10 μm, leaving a non-adhesive circle at the centre; this non-adhesive patch has no effect on our observations and only exists owing to the fabrication protocol (figure 1*a*, electronic supplementary materials). Upon adhesion to the micropatterns, the aggregate localizes to the centre of the pattern and expands a monolayer towards *R*_2_. However, within a margin from *R*_2_, the cells are highly dynamic, with the expansion and retraction of cells within the monolayer. Thus, there is a radius at which there is always a full cell monolayer is always present, 0 < r < *R*_3_, and a region at which the monolayer is transient *R*_3_ < *r* < *R*_2_ (figure 1*b*), where r is the distance from the centre of the micropattern. Cells are imaged in transmitted light (differential interference contrast (DIC)), reflecting an integration through the volume of the aggregate, as well as in fluorescence of the actin (F-tractin) at the surface of the PAA gel, reflecting cell monolayer velocity (figure 1*b*). Simultaneously, we image beads embedded within the PAA gel to be used in traction force microscopy (TFM). We then quantify and compare the dynamics of motion on both unconfined PAA gels and confined PAA gels.

**Figure 2.**
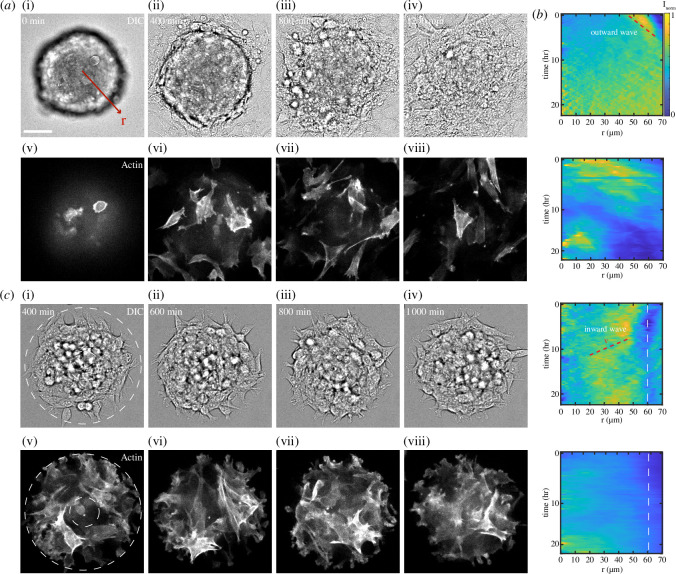
Confining adhesive patterns restrict aggregate spreading. (*a*, top) DIC image of aggregate spreading on a fibronectin-coated PAA gel. r indicates the radial direction in which kymographs are calculated. (*a*, bottom) Fluorescent actin image within the same cells. (*b*, top) DIC image of an aggregate upon a fibronectin micropatterned PAA gel. (*b*, bottom) Fluorescent actin within the same cells. Scale bar is 50 μm. Both PAA gels are Y = 8.6 kPa, incubated in 1 mg ml^−1^ fibronectin. (*c*) Kymograph for normalized intensity, averaged in the radial direction r , for the images in both (*a*) and (*b*). Red-dashed lines indicate regions of inward flow. White-dashed lines indicate $R_{3}$ . The velocity v is indicated and is approximately 15 μm h^−1^. Inward and outward waves are denoted in white text.

In the absence of micropatterns, aggregates spread onto the unconfined PAA gels as has been shown previously (figure 2*a*, electronic supplementary video 1) [36,37,39]. After initial contact between the aggregate and gel, a monolayer emerges from the contact line of the aggregate. Ultimately, the aggregate is depleted, and only a monolayer remains. By contrast, for confined aggregates, spreading stops when the cells reach the micropattern boundary, and the aggregate retains a shallow, ‘dome’ shape (figure 2*b*, electronic supplementary video 2). Using radially averaged kymographs of the DIC and actin images, we observe the radial motion of cells in both confined and unconfined cases (figure 2*c*). While an outward wave is observed for unconfined spreading, multiple radially inward waves are observed in the confined aggregate denoted in figure 2.

### 2.2. Adhesive confinement induces retrograde motions

**Figure 3.**
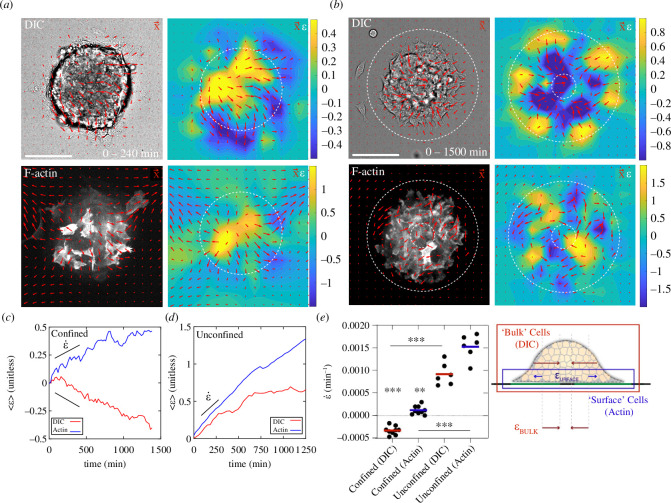
Aggregate adhesive confinement induces retrograde cellular flows. PIV vectors, $\overset{⃑}{X}$ (red arrows) indicating accumulated displacement for confining micropatterns (*a*) and unconfined spreading (*b*), applied to transmitted light images of cells (DIC) and F-actin images at gel surface (F-Tractin). The heat map shows the magnitude of the strain, ε and corresponds to the displacement vectors. Scale bar is 100 μm. (*c*) Strain measured from PIV for confined and (*d*)unconfined/micropatterned conditions. Blue line indicates transmitted light and red line indicates fluorescent actin. (*e*) Strain rate for unconfined and confined conditions. ** Indicates *p*-value < 0.01 and *** indicates *p*-value < 0.001. The diagram that shows net directions for cells moving at the gel surface (blue) versus those moving at the surface and within the bulk (red). N = 30 independent experiments. All vector magnitudes are displayed as normalized to the size of the grid in PIV.

The motion of cells is quantified by particle image velocimetry (PIV), which is applied to DIC and fluorescent actin images (figure 3*a,b*). By transmitted light (DIC), the entirety of the cell aggregate is visible, representing the ‘bulk’ motion of the aggregate. Thus, what is captured by PIV applied to DIC reflects the net motion between what occurs at the gel surface, and what occurs above the gel surface, within the aggregate itself. As there are more cells within the bulk than at the surface, we assume it principally reflects bulk motion. By contrast, F-tractin is visible only within the confocal plane, which is focused on the gel–aggregate surface. Thus, PIV applied to fluorescence images captures the basal or ‘surface’ motion. In both cases, we measure a displacement field, $\overset{⃑}{x}$ , for each point in time. Over time, we accumulate the displacement, $\overset{⃑}{X}=\sum \overset{⃑}{x}$ . From the divergence of the displacement field, we capture the strain of the monolayer and aggregate $\varepsilon =\overset{⃑}{\nabla }∙\overset{⃑}{X}$ , as well as the strain rate, $\dot{\varepsilon }=d\langle \varepsilon \rangle /dt$ which we use to characterize the direction and extent of motion, where $\langle \varepsilon \rangle$ indicates the spatially averaged strain. To reduce the impact of the imaging and detection noises, we also smooth the vector fields (electronic supplementary material, figure S1).

For spreading unconfined aggregates, both the surface and bulk motions are outwards, and $\dot{\varepsilon }$ is positive for both actin and cells (figure 3*a,d*). Furthermore, as the aggregate provides a continuous supply of cells to the monolayer as it spreads, the accumulated strains are large, increasing to over one during the time course of the experiment. By contrast, for confined aggregates, the average strain rate for the surface motion is positive but modest (figure 3*b,c*). This is surprising, as the adhesion area is constant, one may expect a zero-strain rate. However, as this motion is measured purely at the PAA gel surface, a positive strain rate may also suggest that the cells move down towards the gel surface into the monolayer (from the aggregate), move radially outwards along the gel surface, and then up away from the gel surface (towards the aggregate). Indeed, in measuring the motions of the bulk cells in confined aggregates, we find that unlike spreading unconfined aggregates, the strain is no longer positive, but is strongly negative (figure 3*b,e*). There is a persistent inward (‘retrograde’) motion of cells from the periphery to the centre of the aggregate. In combination, an outward motion at the surface, and an inward motion within the bulk suggest persistent cycling of cells towards and away from the PAA gel surface, under the assumption that mass is balanced, and there is not a significant net increase or decrease in the cell population of the aggregate over the timescale of circulation.

### 2.3. The destabilization of protrusions at the adhesive boundary initiates retrograde motions

**Figure 4.**
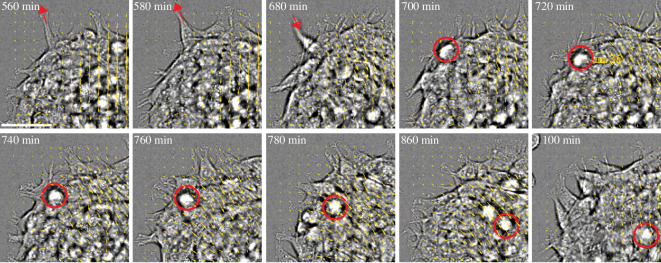
Cell retraction leads to retrograde flow at the micropattern boundary. DIC images of cells within the aggregate (with vector fields overlaid in yellow), where cells protrude (outward red arrow), retract (inward red arrow) and the retracted cell moves towards the centre of the aggregate (red circle). The cell is out-of-plane indicating it has moved upwards into the aggregate. Scale bar is 50 μm.

By monitoring the motion of individual cells, we observe that cells migrate towards the boundary of the micropattern (figure 4). Within approximately 15 μm from the boundary (*R*_3_ <r< *R*_2_), we observe predominantly radially directed cellular protrusions (figure 1*b*). These protrusions are highly dynamic, as they are extended and retracted repeatedly (figure 4, *T* = 680–700 min). This is observed in DIC by the apparent out-of-plane localization of the retracted protrusion. Subsequently, the cell may acquire a more rounded shape (red circle). This out-of-plane rounded shape persists, as the cell moves radially inwards, towards the centre of the aggregate. An out-of-plane cell is indicative of moving upwards, away from the PAA surface. Thus, the inward motion of rounded cells may be suggestive of rearward migration, or their advection, and the influence of large-scale surface stresses. Owing to the rapid, radial and partial motion above the gel surface, we explore the latter possibility. Henceforth, we refer to these bulk motions as ‘flows’ in reference to their resemblance to convective, fluid-like motion. They are differentiated from ‘migration’ which is the motion occurring at the gel surface, driven by substrate traction stress.

### 2.4. The accumulation of traction stress is inhibited on micropatterns

**Figure 5.**
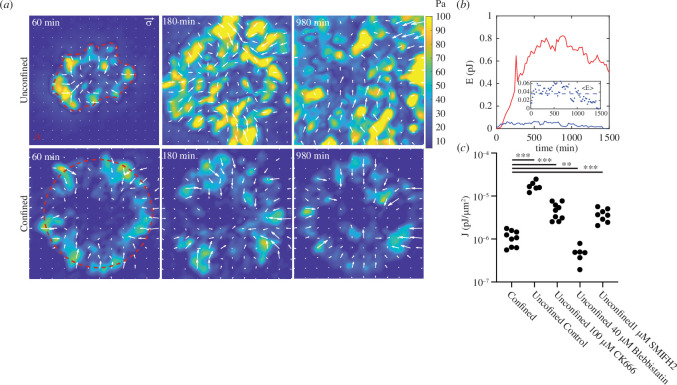
Confinement prevents the accumulation of traction stress. Traction stress ( $\overset{⃑}{\sigma }$ ) for unconfined/spreading (*a*, top) and confined/micropattern (*a*, bottom). The elastic modulus of the PAA gel is Y = 8.6 kPa. Traction force is combined by using two different window sizes: 16 pix (colour map) overlaid with 64 pix (white arrow vector field). A is the contact area between the aggregate and the surface of the gel. (*b*) Strain energy ( E ) for over time for both unconfined and confined conditions. (*b*, inset) Confined strain energy with mean energy, <*E*> indicated. Red-dashed line indicates when the spreading aggregate has reached the boundary of the field of view. (*c*) Strain energy density ( J=E/A ) and mean strain energy for confined and unconfined conditions. *A* is the contact area between the aggregate and the PAA gel. Comparisons are made for where the contact area of the spreading aggregate is roughly equal to the patterned area for the non-spreading aggregate (*A* ~ $A_{0}$ ). ** Indicates *p*-values < 0.01 and *** indicates *p*-values < 0.001. N = 9 (pattern), 6 (control/spreading), 9 (100 μM CK666/spreading), 6 (40 μM blebbistatin/spreading) and 8 (1 μM SMIFH2/spreading) independent experiments. Vector displays as normalized to the size of the grid in PIV.

The motion of cells induces deformations of the PAA gel, which are measured via displacement of the embedded fluorescent microparticles. The displacement is taken between frames, separated by 20 min. From these displacements, the planar strain is calculated, using the divergence of the accumulated displacement field. From the measured strain, we calculate the in-plane traction forces that generate them ( $\overset{⃑}{\sigma }$ ) and the total strain energy, E by TFM (figure 5*a*) [43,44]. This refers to the energy calculated between the deformed configuration for the gel when the cells are attached, to the undeformed configuration after the cells are enzymatically removed. The total strain energy reflects the total mechanical work generated by the aggregate exerted at the surface during motion.

For unconfined spreading, the traction forces ( $\langle \sigma \rangle$ ) increase in time and become elevated at the monolayer boundaries, as has been observed previously [39,41]. The stresses continue to grow as the monolayer expands from the aggregate (figure 5*a*, unconfined). The traction stresses are high throughout the contact area but elevated at the periphery [15]. Ultimately, the magnitude of the peripheral stresses increases to a mean value of ~60 Pa and maximum value of ~200 Pa. By contrast, while the traction stresses in confined aggregates are elevated at the monolayer boundary (micropattern edge), they remain nominal within the contact area. Thus, there is a large decrease in the total mechanical work applied to the substrate. In this case, owing to the inability of the monolayer to expand, the peripheral stresses are greatly reduced, with mean stresses of ~30 Pa and peak stresses of ~100 Pa (figure 5*a*, confined).

The magnitude of traction stresses changes with the contact area between the cells and the substrate. To control for this variation and enable a comparison across a broad range of conditions, we divide the total mechanical strain energy E(pJ) by the contact area $A\left(\mu m^{2}\right)$ to yield an energy density $J\left(\frac{pJ}{\mu m^{2}}\right)$ (figure 5*b,c*). For confined aggregates, the area is equal to the micro-pattered adhesive area ( $A_{0}$ ). For unconfined aggregates, we choose the strain energy that corresponds to a contact area that is equal to the micro-pattered area ( $A\sim A_{0}$ ). Thus, we compare the mechanical energy for equivalent contact areas, between confined aggregates and unconfined aggregates. Consistent with the different distribution of traction stresses, we find that the energy density of unconfined aggregates is approximately 20-fold greater than confined aggregates micropatterned at the same area (figure 5*b,c*). To understand the origins of the decrease in mechanical work, we compare the strain energy density of the confined aggregates to those unconfined but treated with pharmacological agents that influence cellular force generation. To this extent, we compare aggregates spreading treated with 100 μM CK666 that inhibits Arp2/3-based actin polymerization, 1 μM SMIFH2 that inhibits formin-based actin polymerization and 40 μM blebbistatin that inhibits myosin II ATPase activity. In each of these cases, the energy density is decreased in contrast to untreated spreading aggregates. Arp2/3 inhibition decreases the energy density by approximately 73% and formin inhibition by 79% (figure 5*c*). Inhibition of myosin II motor activity had the greatest effect, decreasing the strain energy by 97%. Thus, blebbistatin-treated cell aggregates have strain energies that are most like the strain energies of confined cell aggregates. This suggests that the decrease in traction stresses on micropatterned substrates arises from an inability to accumulate myosin II-generated mechanical work or to transmit the work to the ECM. However, as observed by the strain rates, blebbistatin inhibition does not eliminate migration entirely but can induce aggregate ‘de-wetting’ (electronic supplementary material, figure S2).

### 2.5. Adhesive confinement and mass conservation induce internal circulation

S180 aggregates are viscoelastic, and thus their mechanical properties depend upon the timescales by which stresses or strains are applied. The viscoelasticity depends upon the extent of cell–cell contact, and active stresses generated by the cells. Previously, we and others have characterized the behaviour of S180 cell aggregates as either solid [14] or fluid-like [15,31,45–47] based on the viscoelastic timescale as measured by the micropipette experiment [32]. This timescale was approximately 6 min, and thus, as motion here occurs over hours, we model the aggregate as a viscous fluid. However, we note that our main conclusion that confinement leads to a recirculating flow field is independent of the details of the constitutive laws used to model the dynamics of the aggregate. The recirculation arises owing to the combination of the radially outward monolayer flow, confinement and mass conservation, which lead to the out-of-plane motion of the cells near the edge of the aggregate.

To describe the flow field within the aggregate, we can use the experimentally measured velocity profile of the cell monolayer and impose that as a boundary condition for a viscous drop sitting on the substrate. This introduces ‘activity’ into our model, making it an active drop. The flow field, u=(u,w), in this drop is governed by the incompressible Stokes equations in the polar coordinates ( r , z ) with u representing the radial flow component and w as the vertical component:


(2.1) $$0=-\nabla p+\eta \nabla^{2}u,$$

(2.2) 0=∇⋅u,

where p is the fluid pressure and η is the fluid viscosity. These equations are subject to the boundary conditions *u*( z = 0, r ) = v ( r ), where v ( r ) represents the monolayer velocity and zero shear stress at the droplet interface ∂u/∂z(*z* = *h*) = 0, where h ( r ) represents the height of the drop at a distance r from the centre of the drop. Using the long-wave approximation [48], i.e. assuming the height of the droplet is much smaller than its diameter, we can then obtain the velocity field as:


(2.3) $$u\left(r,z\right)=\frac{1}{2\eta }\frac{\partial p}{\partial r}\left(z^{2}-2hz\right)+v\left(r\right).$$

Here, we have assumed the flow field inside the drop to be axisymmetric; this is a first-order approximation of the real flow field, which is indeed more complex and involves asymmetric contributions. In the case of confined drop, the net flow rate across any arbitrary vertical cross-section within the drop should be zero. We can therefore write the pressure gradient as:


(2.4) $$\frac{\partial p}{\partial r}=\frac{3\eta v\left(r\right)}{h^{2}},$$

indicating that the pressure gradient is created owing to the basal flow in the monolayer.

**Figure 6.**
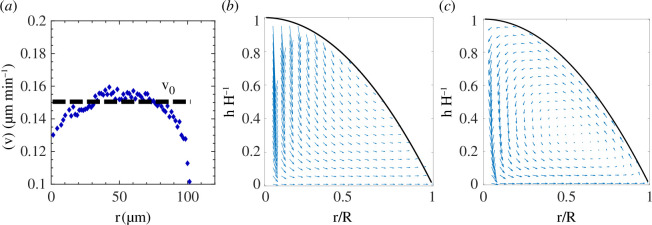
Retrograde bulk flow and anterograde surface flow lead to internal circulation. (*a*) The experimentally measured velocity in the monolayer shows a nearly uniform velocity, which is comparable to the estimated value from the scaling arguments considering the Laplace pressure of the drop to be the driving force for the spreading. (*b*) The monolayer velocity together with the Laplace pressure leads to the spreading of the unconfined aggregate, developing a radially outward flow field. (*c*) The confinement, however, prevents the spreading of the aggregate. Conservation of mass therefore requires the pressure gradients within the drop to adapt to the monolayer-driven flow, leading to circulation within this active droplet.

We can therefore rewrite the flow velocity within the confined aggregate as:


(2.5) $$\frac{u\left(r,z\right)}{v\left(r\right)}=\frac{3}{2}\left({\left(\frac{z}{h}\right)}^{2}-2\left(\frac{z}{h}\right)\right)+1,$$

which recovers u (r,z=0) = v (r) at the base of the drop and shows u ( r ,z = h ) = - v ( r ) /2, i.e. there is a backward flow towards the centre of the drop along the free interface. The normal component of the velocity field w is obtained using the mass conservation in equation (2.2). Assuming a spherical cap shape for the drop, figure 6 shows the recirculating flow field that emerges within the drop owing to this basal flow. Here, we have taken $v\left(r\right)=v_{0}=constant$ as supported by the experimental measurements. This recirculating flow picture is consistent with our experimental observations, suggesting that the basal flow of the monolayer drives a global recirculation within the drop.

At equilibrium, the pressure of a passive drop is determined by the Laplace pressure jump across the interface, i.e. $p=\frac{2\gamma }{R}\sin\left(\theta \right)∼\frac{2\gamma }{R}\theta$ with R and θ≪1 as the drop radius and inner contact angle at equilibrium and γ representing the surface tension of the aggregate. For a liquid drop wetting the substrate, a pressure gradient develops within the drop, the magnitude of which is set by the Laplace pressure jump, i.e. $\frac{\partial p}{\partial r}∼\frac{\gamma }{R^{2}}\theta$ . We would therefore expect the migration speed of the cell monolayer to scale as:


(2.6) $$v_{0}∼\frac{\gamma }{3\eta R^{2}}\theta H^{2}∼\frac{\gamma }{12\eta }\theta^{3},$$

where we have taken H≈Rθ/2 to represent the height of the drop at its centre. Here, we compare aggregates of approximately the same size (*R* = 100 μm), as we have previously reported size-dependent spreading rates [15]. For this size, using the typical values of γ=0.005 N m^−1^ [32], η=2000 Pa ∙ s [15] and θ=π/12 , we find v≈O(0.2) μm min^−1^ , which is comparable to the experimentally measured monolayer velocity (figure 6). In choosing these values, we assume that the surface tension and viscosity do not change, although they are known to change under controlled mechanical perturbations [31].

In the unconfined case, the constraint of zero net flow rate is absent, and both the Laplace pressure gradient and monolayer velocity contribute to setting the flow field within the drop (figure 6). While the total strain energy between confined and unconfined cases is approximately 20-fold different (figure 5*c*), owing to a difference in spatial distribution between the two cases, the mean boundary traction force in the unconfined case is only double that of the confined case for the same radius (electronic supplementary material, figure S3). The difference in the spatial distribution of stress may be owing to the inability to generate stress as the monolayer cannot expand or owing to the non-adhesive centre of the micropattern. Confinement suppresses the traction of cells on the substrate much like how myosin inhibition by blebbistatin reduces the traction on unconfined aggregates.

Alternatively, we can model the monolayer as a two-dimensional active polar fluid, considering cell–cell active contractile and viscous forces as well as cell–substrate friction and traction forces to arrive at the cell monolayer velocity profile (see electronic supplementary material, figure S4). However, we find that one needs to consider viscosities of the order of 2 MPa·s compared with 2 kPa·s used in the scaling arguments above to arrive at velocity magnitudes measured in our experiments. This may be owing to not accounting for the internal flows from the bulk aggregate to the expanding monolayer, which would reduce the effective viscosity of the monolayer itself. However, it should be noted that there are also multiple ways of estimating the viscosity of both spheroids and monolayers that may yield different results. Among these methods include micropipette [31,46], uniaxial compression by parallel plates [29,49–51] and shear rheometry [52]. Differences in viscosity will affect the rate of internal flows, but flows should still exist, as they depend upon the modest positive strain at the surface and confinement at the contact line. Similarly, the assumption of incompressibility is a simplification. For example, changes in cell volume have been observed across spheroids of diverse cell types under applied stresses [33,53]. For S180 cells, we have previously shown that cell volume (density) depends on the spheroid size [32]. Upon the wetting of spheroids to PAA gels as performed here, the density of cells that emerge from the spheroid increases modestly for soft substrates but decreases significantly for rigid substrates [15]. The stiffness used in this study is intermediate between the two. However, the presence/absence of circulation is determined by the monolayer flow and confinement and is independent of the compressibility.

## 3. Discussion

Here, we present a mechanism for how cell migration and convective flows coordinate their dynamics in a simplified model tissue. When aggregates are confined in space, by adhesive micropatterns, the aggregate cannot fully wet the surface. Thus, both the aggregate as well as the monolayer that expands from the aggregate may coexist at a dynamic steady state. At this state, cells migrate along the surface and thus generate traction stresses and subsequently are subject to large-scale stress gradients from the surface of the aggregate. Furthermore, as migration is inhibited at the micropattern boundary, the monolayer cannot expand, which limits the accumulation of migration-based traction stresses. This is owing to the inability of protrusions to stabilize at the boundary. As a result, the protrusion is retracted, and the cell may adopt a rounded shape. Rounded cells are then driven from the periphery of the aggregate to the centre, what we refer to as retrograde flow, owing to its similarity to F-actin flow within the lamella of single cells. Using transmitted light, this motion appears out-of-plane, indicating that the cells have moved above the basal surface, into the bulk of the aggregate. As this motion is out-of-plane, radially directed and opposite the direction of migration at the gel surface, we attribute the origin of this motion to be the conservation of mass, with a contribution from stresses at the surface of the aggregate. To support this point, the surface tension of S180 cell aggregates is measured between 1 and 10 mN m^−1^ [31,32,46]. Furthermore, gradients in surface tension along the surface of approximately 1 mN m^−1^ are sufficient to drive rapid, and long-range internal cellular flows [14,54]. These are referred to as Marangoni flows, although the extent to which they may contribute to aggregate spreading or internal circulationis unclear .

In this study, we assume that the same concentration of fibronectin applied to unpatterned and patterned PAA gels leads to the same friction. However, there may be small differences owing to different methods of coupling adhesive protein to the gels. For non-patterned substrates, fibronectin is coupled to PAA using Sulfo-Sanpah, which covalently links fibronectin to the gel but is itself non-covalently linked within the PAA. By contrast, the same concentration of fibronectin is applied to micropatterns, but linked via EDC chemistry, which is covalent on both ends. Still, the frictional coefficients for aggregate spreading (unconfined) are significantly higher than those on the micropatterns, suggesting fibronectin coupling is not limiting in this case (electronic supplementary material, figure S5).

Confinement of monolayers has been shown to influence the collective motion of cells within the plane [55,56]. In this case, cells exhibit emergent two-dimensional phenomena, such as spontaneous oscillations in their motion, including radial motions and rotations. Relatedly, other works have shown that cells will align and drive chiral flow with respect to their boundaries [57]. In this work, we demonstrate that confinement of three-dimensional aggregate yields emergent phenomena in three dimensions, in which outward (extensile) migration of the monolayer coupled with inward (contractile) flows of the bulk tissue. Thus, autonomous migration and tissue-scale convection coexist to determine dynamic stable behaviours of tissue.

## Data Availability

Data are available in the electronic supplementary material [58].
